# Comparison of 16S rDNA Next Sequencing of Microbiome Communities From Post-scalder and Post-picker Stages in Three Different Commercial Poultry Plants Processing Three Classes of Broilers

**DOI:** 10.3389/fmicb.2019.00972

**Published:** 2019-06-04

**Authors:** Jennifer A. Wages, Kristina M. Feye, Si Hong Park, Sun Ae Kim, Steven C. Ricke

**Affiliations:** Center for Food Safety and Department of Food Science, University of Arkansas, Fayetteville, AR, United States

**Keywords:** microbiome, poultry processing, next generation sequencing, indicator organism, poultry

## Abstract

Poultry processing systems are a complex network of equipment and automation systems that require a proactive approach to monitoring in order to protect the food supply. Process oversight requires the use of multi-hurdle intervention systems to ensure that any undesirable microorganisms are reduced or eliminated by the time the carcasses are processed into final products. In the present study, whole bird carcass rinses (WBCR) collected at the post-scalder and post-picker locations from three different poultry processing facilities (Plant A: mid-weight broiler processing, B: large-weight broiler processing, C: young broiler (Cornish) processing) were subjected to next generation sequencing (NGS) and microbial quantification using direct plating methods to assess the microbial populations present during these stages of the poultry process. The quantification of aerobic plate counts (APC) and Enterobacteriaceae (EB) demonstrated that reductions for these microbial classes were not consistent between the two sampling locations for all facilities, but did not provide a clear picture of what microorganism(s) may be affecting those shifts. With the utilization of NGS, a more complete characterization of the microbial communities present including microorganisms that would not have been identified with the employed direct plating methodologies were identified. Although the foodborne pathogens typically associated with raw poultry, *Salmonella* and *Campylobacter*, were not identified, sequence analysis performed by Quantitative Insights of Microbiology Ecology (QIIME) indicated shifts of *Erwinia, Serratia*, and *Arcobacter*, which are microorganisms closely related to *Salmonella* and *Campylobacter*. Additionally, the presence of *Chryseobacterium* and *Pseudomonas* at both sampling locations and at all three facilities provides evidence that these microorganisms could potentially be utilized to assess the performance of multi-hurdle intervention systems.

## Introduction

The complexity of commercial processing of poultry requires an efficient and extensive network of equipment, automation; and oversight to maintain quality and food safety standards (Chao et al., 2014; Handley et al., 2015; Zweifel et al., 2015). The industry employs a wide range of policies and procedures to control and monitor fecal and ingesta contamination. Upon arrival to the abattoir, live chickens will be dirty, with microorganisms and environmental contamination on their feet, feathers and skin. Furthermore, the nature of slaughter liberates microorganisms from the alimentary tract onto the surface of the carcass and abattoir (Grau, 1986; Rinttilä and Apajalahti, 2013; Oakley et al., 2014; Handley et al., 2015). The dispersal of some microorganisms, namely *Salmonella* and *Campylobacter*, in poultry processing is a constant concern to the industry due to the risk of food borne disease that can be caused by consuming raw poultry (Batz et al., 2012; Buncic and Sofos, 2012).

The scalding and feather removal stages in commercial poultry processing has been identified as one of the first steps within the process with the potential to contaminate poultry carcasses (Dickens and Whittemore, 1997; Berrang et al., 2001; Allen et al., 2003a,b; Buncic and Sofos, 2012). Contamination events have been identified via microbiological culturing techniques including enumeration of indicator microorganism(s) and/or pathogenic microorganism(s), as well as qualitative molecular methods (Corry et al., 2007; Oyarzabal and Hussain, 2010; Oakley et al., 2013). However, the advancements of NGS has allowed for a more comprehensive characterization of the ecology of microbiota on poultry carcasses within the evisceration and chilling stages of poultry processing (Kim et al., 2017; Handley et al., 2018; De Cesare et al., 2018). Analysis of the microbiome profiles of the poultry carcasses sampled at the latter stages of the process have provided information about the efficacy of multi-hurdle interventions, as well as identified both indicator and pathogenic microorganisms impacting food safety and quality (Handley et al., 2018). With this knowledge, it would suffice that the earlier stages of the process, including scalding and feather removal could also benefit from microbiome analysis utilizing NGS technologies. If the microbial communities from these processing stages at different plants are better understood, there could be a possibility to tailor the employment of different antimicrobials based on microbial communities present at the facility level.

This data serves to provide insight into the microbial ecology present within the early stages of poultry processing utilizing both traditional methods and un-restrained-NGS microbiome sequencing. A comparison of traditional microbiological and molecular screens with the unrestrained, culture-free NGS microbiome analysis will determine if weaknesses exist in current methods for the detection of pathogens and indicator organisms. With the application of NGS, a deeper understanding of these shifts will enable the poultry industry to identify new microbiome patterns and indicator organisms, which has the potential to elicit more specific intervention measures and improve food safety.

## Materials and Methods

### Poultry Processing Plant Selection and Operations

Sixty whole bird carcass rinses in total were collected from 3 different commercial poultry plants (*n* = 20) located in the southern geographical region of the United States during the summer months (May to June) of 2015. At each plant, 10 whole bird carcasses were collected from the process line at the post-scalder and post-picker locations. The post-scalder and post-picker locations were chosen for collection sites as these process points represent the early stages of processing and theoretically, carcass rinses performed at these stages would have the highest CFU/mL microbial counts in terms of APC and EB. To limit variation in data, collections at each processing facility were limited to carcasses from the same flock/lot. Sample collection occurred at three different processing facilities, each equipped to process three different classes of broilers. Each class of broiler represented different stages of the grow out period: Cornish hens weighing 2 to 4 lbs. (907 to 1,814 g), mid-weight broilers weighing 4 to 6 lbs. (1,814 to 2,722 g), and larger broilers weighing 6 to 8 lbs. (2,722 to 3,629 g). The poultry processing facilities were chosen to provide microbial data related to bird age, as well as data related to facility specificity as the equipment and operations used are typically tailored to process carcasses of specific sizes as well as the final food product produced.

The commercial processing systems at each facility had similar scalding and picker operations. All facilities had scalder tanks equipped with a counter-flow water influx in which fresh water was added to the final tank. Tank overflow was released from the initial tank, which is where the organic load would be expected to be the highest. This allows carcasses to move through progressively cleaner water in the scalder tanks. The water temperature and immersion time varied slightly for each facility due to the size of the bird and its end consumer use. Plant A and B operated a soft scald system in which carcasses were immersed in a 52 to 57°C scalding tank for 180 s. Plant C also operated a soft scald system, however, as the carcasses were smaller, the dwell time was slightly reduced to between 90 to 100 s. After scalding, the carcasses moved into the feather removal or picking machines where rubber fingers or protrusions applied pressure to pull and remove feathers from the follicles. The operations of the picking machines were similar for all three facilities, in which the carcasses were subjected to three banks (each 0.91 meter in length) of rubber fingers. Total picking time averaged 180 s for each carcass.

### Sample Collection

Conforming to USDA protocols (USDA, 2013), whole carcasses were randomly selected from the operating lines at the designated sampling points (post-scalder and post-picker) and placed in sterile rinse bags. Carcasses were selected in quick succession at both locations and samples were representative of the same lot/flock. After collection, 400 mL of Butterfield’s Phosphate Buffer solution were poured over the surface of the carcass and the rinse bag was folded close. The carcasses were rinsed for 1 min in an arcing motion to ensure that the rinsate could move along the carcass surface. The collected rinsate was transferred to the original container and these containers were placed on ice in a cooler and then transported to the testing laboratory.

### APC and Enterobacteriaceae Analyses

Upon receipt at the testing laboratory, 1.0 mL aliquots of rinsates and associated serial dilutions were enumerated for APC and EB counts with 3M^TM^ APC or EB count petrifilm^TM^ (3M^TM^, St. Paul, MN, United States). The gelling and nutrient components of 3M^TM^ Petrifilm^TM^ allow for the analysis and enumeration of a wide range of microorganisms. Analyses for APC counts were performed per Official Methods of Analysis (OMA) 990.12 published by the International Association of Official Analytical Chemists (AOAC) and analysis for EB counts were performed according OMA 2003.01 with a modified incubation temperature (35 ± 1°C) per Compendium of Methods for the Microbiological Examination of Foods recommendations (Moberg and Kornacki, 2013).

### 16S rDNA Microbiome Sequencing

Aliquots (50 mL) of the rinsates were centrifuged (Sorvall Lynx 6000, Thermo Fisher Scientific, Langenselbold, Germany) for 15 min at 8,000 g, to pellet the bacterial cells. Genomic DNA extraction of the formed pellets were performed using a QIAamp DNA Stool Mini Kit (Qiagen, Valencia, CA, United States) following the kit’s standard protocol except for reducing the elution volume to 50 μL. The DNA concentration and purity were measured for each extraction using a NanoDrop ND-1000 (Thermo Scientific, Wilmington, DE, United States). Aliquots were stored -20°C until further analysis could be performed.

The sequencing pipeline is as per Kozich et al. (2013) and Park et al. (2016). The individual rinsate sample DNA was diluted to 10 ng/μL and an Illumina MiSeq Library was prepared, and targeted the V4 region of 16S rRNA using dual-indexed primers via Eppendorf Mastercycler pro S (Eppendorf, Westbury, NY, United States). Confirmation of amplicon presence and relative size was conducted via a 1% agarose gel. The library was normalized via SequalPrep^TM^ Normalization kit (Life Technology) as per the manufacturer’s instructions, except that the final elution volume was modified to 15 μl. The library was pooled and assessed for purity and concentration was evaluated via quantitative polymerase chain reaction (qPCR) using the standard protocol from the KAPA Library Quantification Kit (Kapa Biosystems, Woburn, MA, United States). The qPCR reactions were performed on an Eppendorf Mastercycler EP Gradient S (Eppendorf, Westbury, NY, United States) (*R*^2^ = 0.999; Efficiency = 0.97). The amplicon size and concentration was further confirmed by Agilent 2100 Bioanalyzer System (Agilent, Santa Clara, CA, United States). Amplicons were diluted to 4 nM and combined with prepared PhiX Control v3 (5%, v/v) (Illumina, San Diego, CA, United States) and sequenced. Sequencing was carried out as per the Illumina MiSeq v2 (500 cycle) Reagent Cartridge (Illumina) instructions.

### QIIME Sequence Data Processing and Analysis

The sequence output (FASTA files) was downloaded from the Illumina Biospace Website and the following protocol is based on Park et al. (2016). The analysis of the sequence reads was performed using Quantitative Insights into Microbial Ecology (QIIME) pipeline version 1.9.0, which provides analysis of the sequence, classification of Operational Taxonomic Units (OTUs), and species diversity and richness of the reads. Sequences were assigned to taxonomic levels based on 97% identity levels to those found on the Greengenes 16S rRNA gene database and chimeric sequences were discarded. Samples for each facility (Plant A, B, and C) were normalized to a fixed read depth based on the lowest number of sequences achieved for that sample set. Associated alpha and beta diversity measurements were obtained from QIIME package using these read depths. Taxonomic tables of sequences were limited to sequences present at relative abundances greater than or equal to 0.5% within that facility’s sample set.

### Statistical Analysis

Enumerated counts (CFU/mL) obtained from 3M^TM^ petrifilm^TM^ analyses for each carcass were log_10_ transformed. The log_10_ transformed average values were compared for mean differences in microbial populations between post-scalder and post-picker sample collections within each facility, and used to provide numerical values for comparisons among the three processing facilities. The log transformed count data were evaluated using an analysis of variance (ANOVA) in SAS (Statistical Analysis Software, Cary, NC, United States), where collection site was treated as a main effect and location means were separated using Duncan’s multiple range test. Statistical significance is determined at *p* < 0.05.

Alpha and beta diversity calculations were used to analyze the resulting OTU sequences. Alpha diversity measurements including Chao 1 indices and OTU rarefaction curves, provided information about the OTU richness and diversity of individual samples within each population. Beta diversity measurements including both weighted and unweighted UniFrac diversity plots of principal coordinate analysis (PCoA) (Lozupone and Knight, 2005) were used to evaluate OTU diversity between the post-scalder and post-picker populations. Utilizing these diversity measurements, the microbiomes were analyzed to determine what differences existed in sampling locations (post-scalder and post-picker) for each of the facilities.

## Results

### APC and Enterobacteriaceae Load Quantitation

**FIGURE 1:**
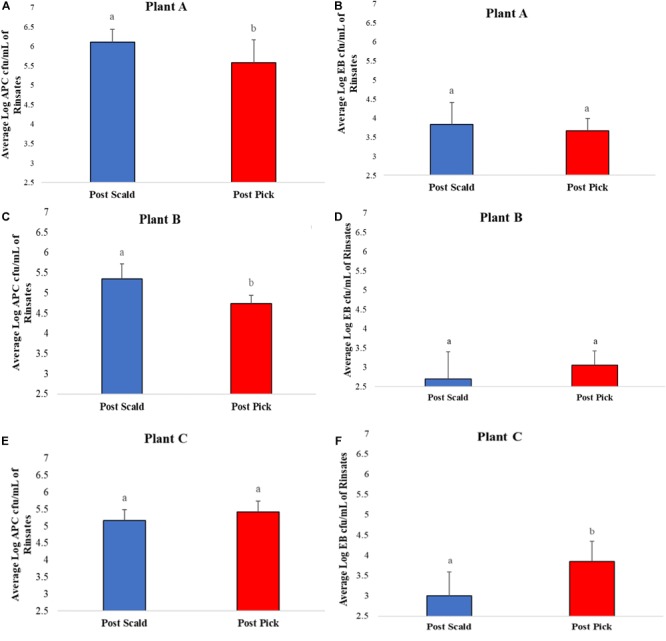
Average Log APC **(A,C,E)** and EB **(B,D,F)** counts of rinsates collected at post-scalder and post-picker location for Plants A, B, and C. ^ab^Log values denoted with different letters were significantly different between the post-scalder and post-picker collection sites at *P* ≤ 0.05.

Average bacterial counts of APC and EB on chicken carcass rinsates from post-scalder and post-picker at three different processing plants are presented in Figure 1. The average log APC counts of rinsates from the post-picker location at Plant A and Plant B were 0.53 log CFU/mL and 0.61 log CFU/mL lower, respectively, and significantly different (*p* ≤ 0.05) than the rinsates collected from the post-scalder locations (Figure 1A,C). In contrast, there was no significant difference in APC levels between post-scalder and post-picker location at plant C (Figure 1E). At Plant C, EB counts for the post-picker rinsates were 0.84 log CFU/mL higher than rinsates from the post-scalder location (Figure 1F). While the increase in EB counts between the two sampling locations at Plant C was significantly different (*p* ≤ 0.05), there were no significant differences in EB levels from plant A and B (Figure 1B,D).

### Microbiome Analysis

**FIGURE 2:**
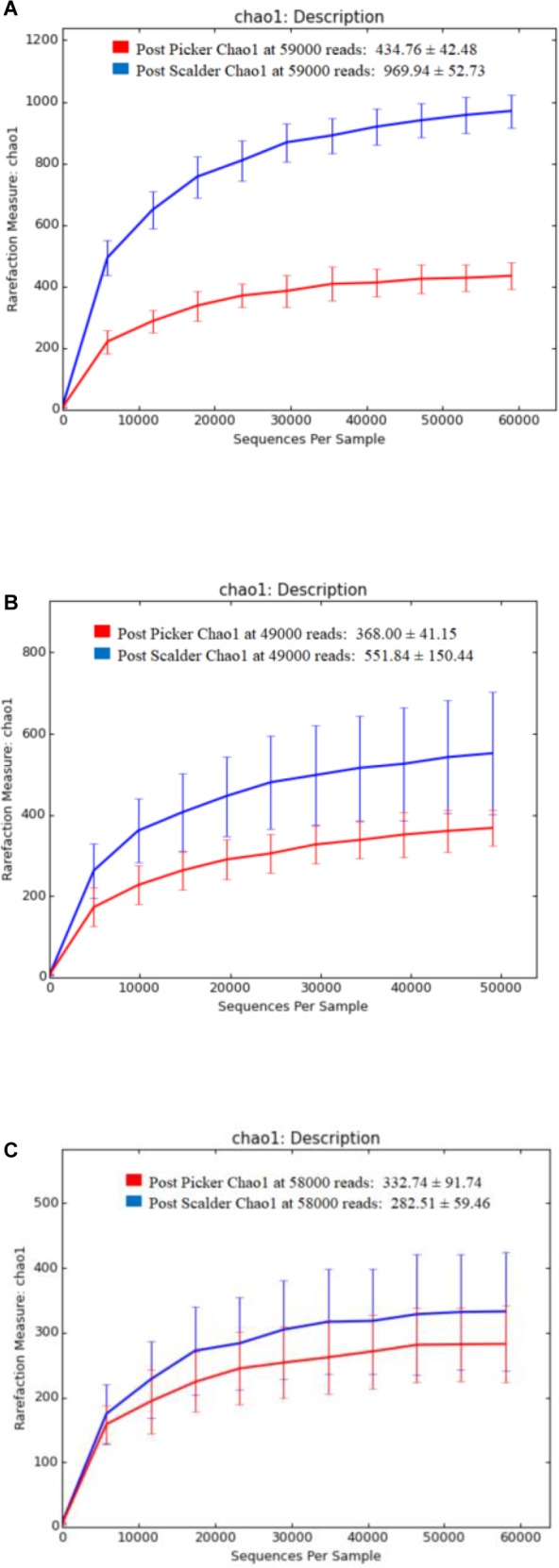
Chao 1 alpha diversity metrics for sampling locations within each facility. **(A)** represents richness measurements for Plant A OTUs. **(B)** represents richness measurements for Plant B OTUs. **(C)** represents richness measurements for Plant C OTUs.

**FIGURE 3:**
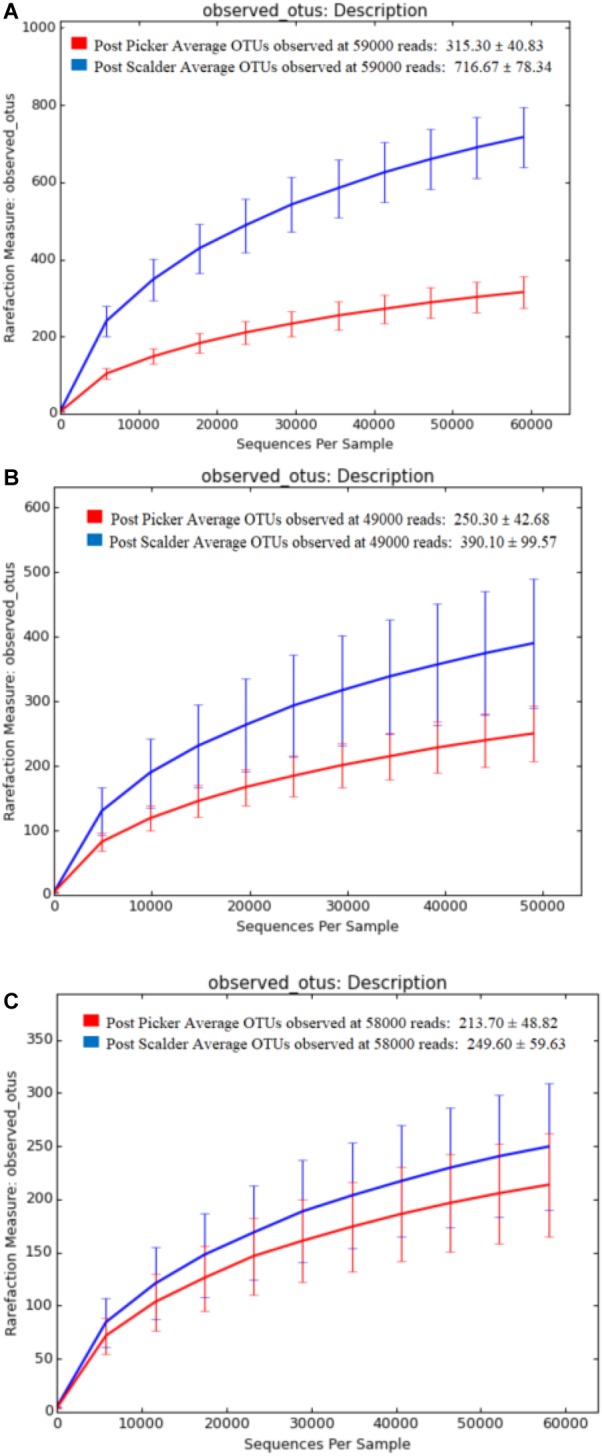
OTU rarefaction curves depicting number of OTUs versus sequence depth for sampling locations within each facility. **(A)** represents Plant A observed OTUs. **(B)** represents Plant B observed OTUs. **(C)** represents Plant C observed OTUs.

Alpha and beta diversity are important tools that are utilized as a component of microbiome analysis to assess the diversity of sequencing depth (alpha) and the compositional variety between samples (beta). Normalization of the OTU sequences that were analyzed per facility, ensured that the depth of sequences was equal and allowed for alpha diversity measurements via Chao1 and OTU rarefaction curves (Figure 2, 3, respectively). Overall, the samples collected at the post-picker site were generally less diverse in terms of OTU richness when compared to the samples collected at the post-scalder locations. The largest difference between the two sampling locations was observed at Plant C, while the smallest difference was observed at Plant B. Observed OTU rarefactions indicated there were lower numbers of OTUs observed in samples collected at the post-picker locations when compared to those samples collected at the post-scalder locations. The largest number of OTUs for both sampling locations was observed at Plant A.

**FIGURE 4:**
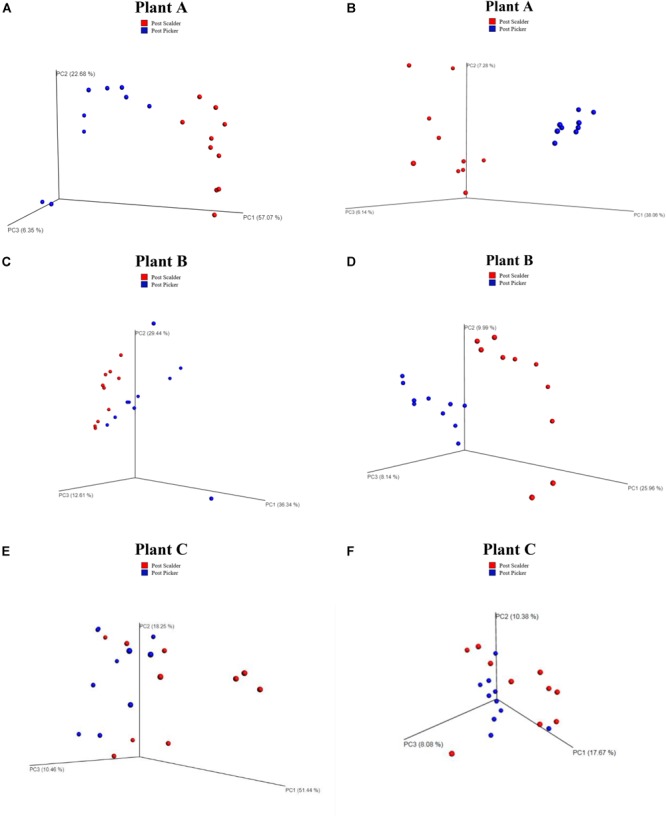
Weighted and Unweighted UniFrac plots depicting OTU diversity between microbiome populations of rinsates collected at post-scalder and post-picker locations. Weighted plots consider the relative abundance values of the OTUs present, and unweighted plots are based on the number of unique OTUs. Weighted UniFrac plots for Plant A, B, and C are represented in left column **(A,C,E)**, respectively. Unweighted UniFrac plots for Plants A, B, and C are represented in right column **(B,D,F)**, respectively.

Weighted and unweighted principal coordinated analysis (PCoA) UniFrac plots generated by the beta diversity analysis from the three plants are presented in Figure 4. Beta diversity measurements from Plant A demonstrated clustering based on location for both the weighted and unweighted PCoA plots. Comparing the two PCoA plots, the communities appear to have mostly different compositions, although clustering is somewhat reduced in the weighted plots which suggest the populations are slightly more similar than what was depicted by the unweighted measurements. Conversely, for Plants B and C, the weighted PCoA plots do not demonstrate distinct separation between the two communities, whereas the unweighted PCoA plot does demonstrate clustering based on sample location, although the separation is slightly more pronounced at Plant B.

**FIGURE 5:**
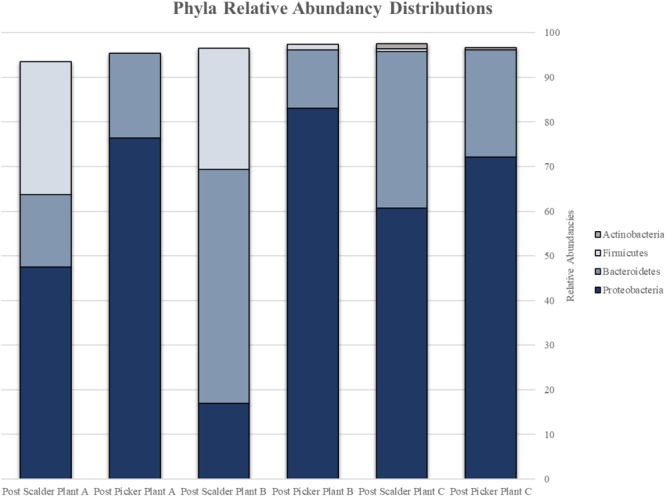
Relative abundancies of phyla represented by OTUs present at post-scalder and post-picker sites for Plant A, B, and C. OTUs at Plants A and B were representative of *Proteobacteria, Bacteroidetes*, and *Firmicute*s. OTUs at Plant C were representative of *Proteobacteria, Bacteroidetes, Firmicutes*, and *Actinobacteria*.

**FIGURE 6:**
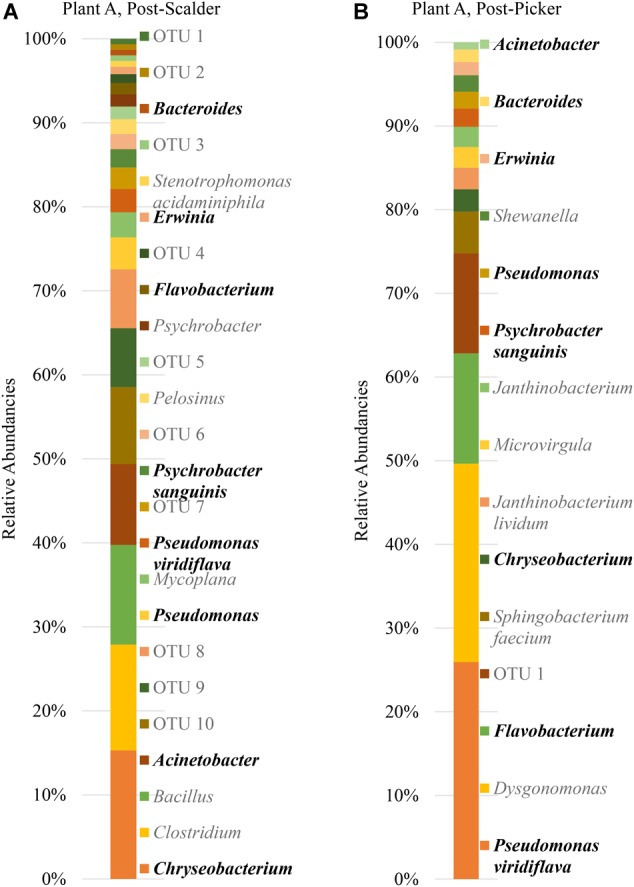
Relative abundancies of OTUs at Plant A. **(A)** Post-scalder collection site. **(B)** Post-picker collection site. OTUs in bold were present at both sampling locations.

**FIGURE 7:**
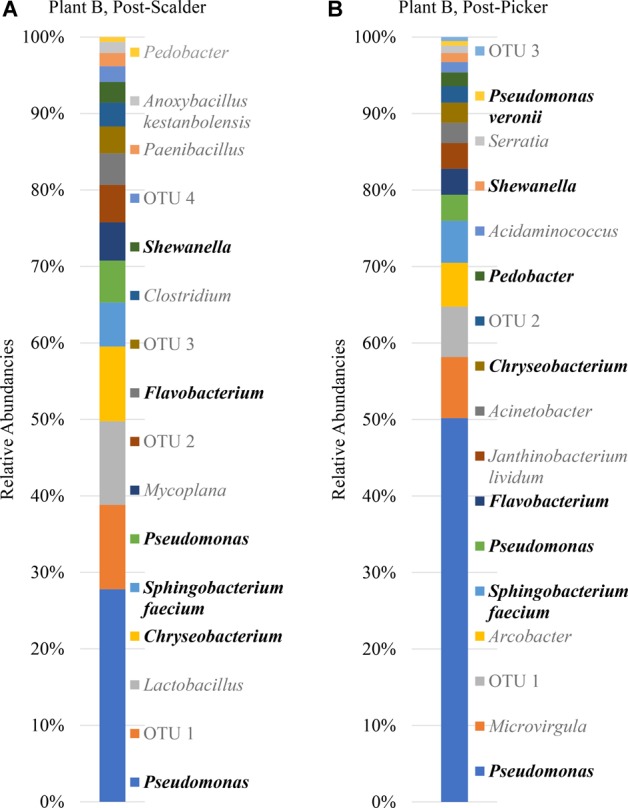
Relative abundancies of OTUs at Plant B. **(A)** Post-scalder collection site. **(B)** Post-picker collection site. OTUs in bold were present at both sampling locations.

**FIGURE 8:**
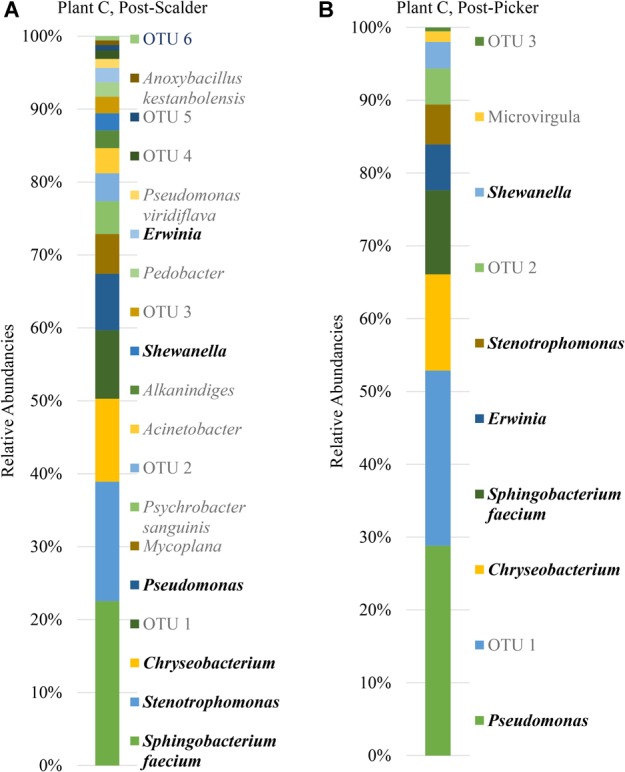
Relative abundancies of OTUs at Plant C. **(A)** Post-scalder collection site. **(B)** Post-picker collection site. OTUs in bold were present at both sampling locations.

The major bacterial phyla represented in the pooled rinsates are presented in Figure 5, and the major genera identified within the pooled rinsates from each facility are presented in Figure 6–8. Samples collected at Plant A at the post-scalder location had the largest number of OTUs present with most the OTUs being derived from three major phyla groups: *Firmicutes, Proteobacteria*, and *Bacteroidetes*. At the post-picker location for Plant A, no OTUs represented the phyla *Firmicutes*, but there was a large increase in relative abundance of phyla *Proteobacteria* (47.50 to 74.65%), and a small increase in phyla *Bacteroidetes* (16.19 to 18.89%). The most abundant sequences (29.74%) within the phyla *Proteobacteria* belong to the *Pseudomonadaceae*, and 24.73% are identified to genus level as *Pseudomonas*, and 4.74% are identified to species level as *Pseudomonas viridiflava* (Figure 6). There was also an increase in OTUs representing the phyla *Bacteroidetes*, and furthermore, post-picker samples appeared to be more diverse as there were five OTUs identified (*Flavobacterium, Chryseobacterium, Sphingobacterium, Dysgonomonas*, and *Bacteroides*) while only three OTUs were identified (*Flavobacterium, Chryseobacterium*, and *Bacteroides*) in post-scalder samples (Figure 6).

Plant B relative abundance values of OTUs were like Plant A, with the exception that OTUs belonging to phyla *Firmicutes* were still present at the post-picker location, albeit at low levels (1.31%) (Figure 7). This is interesting as the relative abundance levels (16.41%) of *Firmicutes* at the post-scalder location were lower than those observed within Plant A levels (29.85%). Like Plant A, the relative abundance levels of *Proteobacteria* at the post-picker location (83.12%) were higher than the relative abundance levels of *Proteobacteria* at the post-scalder location (59.47%). For both sampling locations, the most abundant sequences from the phyla of *Proteobacteria* were from *Pseudomonadaceae* (31.67 and 52.76%, post- scalder and post-picker, respectively), although none of the sequences are identified past the genus level at the post-scalder site, and only one species was identified at the post-picker site (*Pseudomonas veronii*) (Figure 7).

At Plant B, there was an increase in relative abundance values of OTUs identified as belonging to *Enterobacteriaceae* between the two sampling locations (1.96 and 3.04%, post- scalder and post-picker, respectively). At the post-picker location, the *Enterobacteriaceae* was represented by *Serratia* (0.95%) and an unidentified sequence (2.09%). At the post-scalder location, none of the sequences were representative of *Campylobacteraceae*, while 5.59% of the OTUs present at the post-picker location could be attributed to *Campylobacteraceae*, identified as *Arcobacter.* While there was an increase in *Proteobacteria* OTUs between the two sampling locations, the relative abundance level of phyla *Bacteroidetes* decreased (19.32 and 12.96%, post-scalder and post-picker, respectively), although the genera diversity between the two sites appears to be similar as the same OTUs were present in both locations (*Flavobacterium, Chryseobacterium, Pedobacter*, and *Sphingobacterium*).

Many of the OTUs obtained from both sampling locations at Plant C represent microorganisms belonging to phyla *Proteobacteria* and *Bacteroidetes*, although there were a small percentage of members of phyla *Firmicutes* and *Actinobacteria* present at the post-scalder location. The relative abundance of *Proteobacteria* OTUs at each sampling location increased from 60.75 to 72.19%. The most abundant OTUs from the *Proteobacteria* phyla were identified as *Stenotrophomonas* (15.96%) at the post-scalder location, and *Pseudomonas* (27.87%) at the post-picker location (Figure 8). Like the other plants, there was an increase in the relative abundance of OTUs belonging to *Enterobacteriaceae* (represented by genus *Erwinia*) between the two sampling locations (1.92 and 6.07%, post-scalder and post-picker, respectively). At Plants A and B, there was a decrease in *Bacteroidetes* OTUs, between the two sampling locations (35.09 and 23.96%, post-scalder and post-picker, respectively), as well as a decrease in the genera diversity as only two of the three genera present at the post-scalder location (*Chryseobacterium, Pedobacter*, and *Sphingobacterium*) were present at the post-picker location (*Chryseobacterium* and *Sphingobacterium*).

## Discussion

Traditional microbiological methods have been employed to detect pathogen and indicator organism carriage and load on rinsates. Interestingly, the same methods have also detected the differences in the growth conditions, transport and processing facility environments, and bird age at processing (Grau, 1986; Lyon et al., 1991; Davies and Wray, 1994; Davies, 2005; Zweifel and Stephan, 2012; Rinttilä and Apajalahti, 2013; Oakley et al., 2014; Schilling et al., 2014; Kim et al., 2017). This research uses these methods as well as microbiota data to assess and quantify differences between microbial loads on different sized carcasses before and after feather removal (picking) to potentially provide a more robust microbiological profile of these process stages at three different processing facilities.

The age of the broiler, and the specificities of equipment and operations within the processing facility undoubtedly play roles in creating microbial ecology diversity. Understanding how the microbiomes are established as well as how they differ between facilities could be used by processors to tailor intervention strategies on a plant by plant basis. Further, the identification and monitoring of closely related microorganisms belonging to the same family as the target pathogen(s) can provide a more robust indication of the risk of those pathogens in the process or system being evaluated. Particularly, for this data set, shifts of phyla *Proteobacteria*, which encompass OTUs from *Enterobacteriaceae* and *Campylobacteraceae*, could be used to determine how these process steps influence the presence or persistence of those microorganisms that can cause food borne illnesses, namely *Salmonella* and *Campylobacter*. Determining the most inclusive indicator microorganism(s) is dependent on several factors including whether the microorganism(s) are adequate in terms of their response to the environment and conditions that the target pathogen(s) encounter (Sinclair et al., 2012). Response of microorganisms in any given environment is controlled by the genetic makeup, and taxonomically related microorganisms will typically respond similarly (Sinclair et al., 2012), so close evaluation of related microorganism(s) present within a system could be informative when microbiologically evaluating a process or system. At Plant A, OTU composition of samples collected at the post-scalder site demonstrated a relatively large proportion of the phyla group *Proteobacteria* (47.50%), although only 0.81% of the OTUs represented *Enterobacteriaceae*, of which *Salmonella* belong. The *Enterobacteriaceae* present were instead identified as belonging to the genus *Erwinia* (Figure 6). Similar results from plants B and C also indicated increases as well as changes in OTU composition of phyla *Proteobacteria* abundancies between the two sampling locations. At Plant B, OTUs representing *Enterobacteriaceae* at the post-scalder location (unidentified OTU) were different than those identified at the post-picker location (unidentified OTU and *Serratia*) and at Plant C, *Enterobacteriaceae* OTUs present were identified as genus *Erwinia*. None of the sequences represented OTUs from *Campylobacteraceae*, of which *Campylobacter* belong, although 5.59% of the sequences identified at the post-picker location at Plant B were identified as genus *Arcobacter*. *Arcobacter*, previously identified as “aerotolerant Campylobacters” (Phillips, 2001), can grow at colder temperatures when compared to *Campylobacter* sp., and have also been isolated from human feces in patients presenting with intestinal distress (Atabay et al., 1998; Kabeya et al., 2004). *Arcobacter* has been isolated at various rates in broiler meat, fecal and cloacal sampling (Kabeya et al., 2004), but the rates of isolation in poultry carcasses compared to those obtained from caecal sampling indicate that carcass contamination is a function of the processing environment and not necessarily due to infection present within the entire flock (Phillips, 2001; Driessche and Houf, 2007). As the processing steps evaluated in this study occur prior to any evisceration steps, the presence of microorganisms belonging to *Enterobacteriaceae* indicate that the enteric microorganisms are present within the scald waters and associated equipment surfaces and could likely be attributed to expulsion of the viscera as well as external contamination of the bird’s feet and feathers. The persistence of these microorganisms in the environment could also be a factor of their abilities to adhere to various surfaces including stainless steel which could allow for their survival in process waters (Assanta et al., 2002; Driessche and Houf, 2007).

The presence and increase in relative abundance of the phyla *Bacteroidetes* within this sample set may also be an indication of the effect the processing environment has on possible cross contamination events within these stages. Members of this phyla, have been identified as major constituents in the microbial consortium of the lower GIT of poultry (Han et al., 2016; Oakley and Kogut, 2016). Similar to findings by Handley et al. (2018), the presence of *Chryseobacterium* at both post-scalder and post-picker sites, which occur prior to an evisceration steps, indicate that this microorganism could be indicative of cross-contamination due to presence of fecal material and soil on the feathers and feet of processed carcasses. Isolation and identification of this class of microorganisms could therefore be useful in evaluating processing systems to better understand the impact of fecal material, soil, and feather contamination as well as provide information regarding the extent of cross contamination events that may occur between flocks.

It is well documented that species belonging to Pseudomonadaceae are often isolated from raw and spoiled poultry products (Nychas et al., 2008; Deusch et al., 2015). Pseudomonadaceae are introduced into the processing environment on the feet, feathers, dirt and debris of the birds being processed, and the associated sterile muscle meat produced becomes contaminated with these spoilage microorganisms via equipment, water and aerosol production in the processing environment (Geornaras et al., 1999; Ahmet et al., 2015; Rouger et al., 2017). Understanding the microbiome of these poultry process stages can help to determine shifts in Pseudomonadaceae populations including when the microorganisms are introduced, dispersal routes, as well as response to sanitation practices and environment changes. While some of these shifts may be identified by direct plating techniques, including 3M^TM^ petrifilm^TM^, the increase of spoilage organisms represented by Pseudomonadaceae may not be specifically identified due to the selectivity induced by the mesophilic incubation temperatures used for the petrifilm analyses (Barnes, 1972; Oyarzabal and Hussain, 2010). Moreover, as biofilm formation of some *Salmonella* species has shown to be enhanced in the presence of *Pseudomonas*, understanding the effect of environment changes on Pseudomonadaceae could aid in understanding the effects that those changes may also exert on *Salmonella* (Habimana et al., 2010). Specifically, in this data set, the dominance of species from the *Pseudomonadaceae* could represent an opportunity to address interventions/measures that could better control the presence and proliferation of both spoilage and pathogenic microorganisms that may be present. The detection of *Pseudomonas* within different stages of the processing system serves to illustrate that these microorganisms could also be used as indicators of process performance as suggested previously by Handley et al. (2018).

## Conclusion

This study is a unique approach to the application of microbiome analysis of the earliest, and microbial diverse stages (scalding and feather removal) within a commercial poultry processing system. Moreover, it highlights how diversity can differ among facilities, and that this diversity may change depending on the facility’s equipment designs and operational standards as well as the age of the birds. Although, *Salmonella* and *Campylobacter* OTUs were not identified, the identification of closely related genera including *Erwinia, Serratia*, and *Arcobacter* could indicate that these stages could be further evaluated for possible improvements in terms of reducing *Enterobacteriaceae* microorganisms, which could help to reduce incidence of these microorganisms in later stages of the processing systems. Specifically, the presence of genus *Erwinia* at both post-scalder and post-picker locations at Plants A and C could indicate that these microorganisms could be used as predictors of *Enterobacteriaceae* prevalence and persistence in the environment which could provide information about potential carcass and flock cross-contamination events. Additionally, the differences in OTU populations between post-scalder and post-picker locations for all three plants could indicate that the sources of the OTUs differed between the two locations, and specifically increases in diversity on post-picker carcasses could be attributed to the processing environment being influenced by other carcasses or flocks being processed on the same day. While this is somewhat expected, microbiome analysis provides a deeper understanding of the complete microbiome present, and provides specific identities of microorganisms to help determine the extent of the microbial cross contamination between these two stages within the process. Repeating this experiment and considering flock or shift variations could be used to help identify and evaluate the risks within these and other processing stages.

## Author Contributions

JW, SR, and SP conceived the experiment. JW executed the experiment. JW, SP, KF, and SK analyzed the data. JW, KF, SR, and SK prepared the manuscript. KF and JW prepared the final data.

## Conflict of Interest Statement

The authors declare that the research was conducted in the absence of any commercial or financial relationships that could be construed as a potential conflict of interest.
